# Novel Pharmacological Therapy in Inflammatory Bowel Diseases: Beyond Anti-Tumor Necrosis Factor

**DOI:** 10.3389/fphar.2019.00671

**Published:** 2019-06-18

**Authors:** Cristiano Pagnini, Theresa T. Pizarro, Fabio Cominelli

**Affiliations:** ^1^Department of Gastroenterology and Digestive Endoscopy, S. Giovanni Addolorata Hospital, Rome, Italy; ^2^Department of Medicine and Pathology, Case Western Reserve University, Digestive Health Institute, University Hospitals of Cleveland, Cleveland, OH, United States

**Keywords:** inflammatory bowel disease, Crohn’s disease, ulcerative colitis, therapy, biologics, oral small molecules

## Abstract

Inflammatory bowel diseases (IBDs) are chronic conditions of the gastrointestinal tract in which dysregulated immune responses cause persistent inflammation of the gut mucosa. Biologic therapy with anti-TNF blockers has revolutionized the therapeutic management of IBD for their remarkable efficacy and potential impact on disease course and for many years has represented the sole treatment option for patients refractory or intolerant to conventional therapy. In recent years, more molecules, both biologically and chemically synthetized, have been developed as potential therapeutic options for IBD that target different molecular pathways aside from TNF blockade, and which have been proposed as targets for novel drugs. This is particularly relevant for the present, as well as future, management of IBD, considering that some patients are refractory to anti-TNF. This review will summarize the pharmacological options, either currently available or in the pipeline, for market approval to treat IBD, besides anti-TNF strategies, based on their mechanism(s) of action. We will also analyze the current evidence for effectiveness and safety, as well as offer perspective, regarding the potential implementation for such therapies in the future.

## Introduction

Inflammatory bowel disease (IBD) is a condition involving the gastrointestinal tract that displays a chronic remittent clinical course, with alternating bouts of remission and flares of active inflammation. The etiology of IBD remains unknown, but its pathogenesis is associated with dysregulated immune responses that drives a persistent inflammatory state within the intestinal mucosa. Crohn’s disease (CD) and ulcerative colitis (UC) are the two main entities of IBD, which each presents particular clinical and anatomo-pathological features (Abraham and Cho, 2009). Specifically, CD is characterized by transmural inflammation that typically involves all layers of the gut wall, is discontinuous and patchy in appearance with alternating affected and non-affected areas, and can affect the entire gastrointestinal tract, from mouth to anus (Baumgart and Sandborn, 2012). UC, on the other hand, affects the most superficial mucosal layer of the gut wall, which usually arises from the anus and continuously extends proximally to variable degrees throughout the whole colon (Ordás et al., 2012). Both CD and UC display specific differences, but both conditions represent challenges for patients and physicians, and are considered disabling diseases. As such, therapeutic strategies to treat IBD have changed throughout the years, shifting from solely resolving disease symptoms to profound healing of the intestine, with the end result of not only treating short-term complications, but also impacting the natural history of disease by reducing important outcomes, including hospitalization and surgery (Neurath and Travis, 2012). Biologics have been at the forefront of this change. In fact, biologic (or biotechnologic) drugs are molecules that, differently from “classic” or chemical drugs, are produced by biologic systems and target specific molecules or pathways involved in the inflammatory cascade that is triggered during IBD. They are characterized by consistent efficacy, rigorously evaluated by pre-clinical and phase II/III clinical studies, and are generally indicated for moderate-to-severe IBD patients that are not responding or are intolerant to conventional therapies. Biologics are generally large molecules (e.g., monoclonal antibodies) that require parenteral administration and are characterized by a variable degree of immunization.

The prototypic biologic drug is infliximab, a chimeric anti-TNF antibody that appeared on the market for the treatment of IBD in the late 1990s. From that time until just a few years ago, the anti-TNF blockers (e.g., infliximab, adalimumab, certolizumab pegol, and golimumab) have been the only approved biologic drugs for the treatment of IBD, with the exception of natalizumab, which has been available only in the U.S., under specific restrictions due to its safety profile (Pagnini et al., 2017). These drugs, which remain the gold standard to treat moderate-to-severe IBD, have displayed a consistent response rate for the induction and maintenance of disease remission. Moreover, they have shown dependable efficacy for not only healing of the gut mucosa and relief of symptoms, but also reduced hospital admission rates and improved quality of life for IBD patients (Van Assche et al., 2010). Nonetheless, a consistent subset of patients (around 20%) do not respond to treatment, and a similar proportion of patients is likely to lose efficacy every year (Wong and Cross, 2017). Although these drugs are generally considered safe, adverse events are still not infrequent and some patients present contraindications (Pagnini et al., 2015). Thus, considering the aforementioned issues, and the fact that the proportion of patients who have already experienced anti-TNF therapy is constantly increasing, the development of different biologic drugs with alternative mechanism(s) of action has become an urgent need for the treatment of IBD. Besides biologic drugs, a new generation of chemically synthetized oral small molecules have also been recently developed. In fact, oral administration is generally better accepted by patients, does not confer costly and time-consuming infusions in a hospital setting, and generally guarantees a lower risk of immunization. On the other hand, serum concentrations of the drug are less tightly regulated, and the absorption of the compound may be more variable and affected by active inflammation or a resected bowel (Vetter and Neurath, 2017). At present, new biological and chemical drugs have recently been emerging on the market, and more molecules are yet under way for approval. The focus of this review is to summarize the present and upcoming new drugs, aside from TNF blockers, for the treatment of IBD according to their mechanism of action(s).

## Drugs Interfering With Leukocyte Trafficking

The first pathway to be investigated for therapeutic intervention, aside from TNF blockade, was to target leukocyte trafficking, which is a process that includes extravasation and priming of cells from the vasculature, migration and homing of activated cells into the intestinal tissues, and retention and egress of leukocytes within the gut mucosa (Zundler and Neurath, 2017). Specifically, the infiltration of lymphocytes (e.g., T-cells) into the intestinal mucosa represents a target for molecular intervention for therapeutic purposes in IBD. Leukocyte adhesion and extravasation is a multi-step process that includes the tethering/rolling, activation, adhesion, and extravasation/migration of leukocytes into the intestinal mucosa. This complex process is characterized by low-afﬁnity bonds between integrins on the surface of circulating lymphocytes and their inducible ligands located in intestinal cells of the endothelium, interaction that induces a rolling and adhesion effect, with the result of a slowing down of the circulating leukocyte. Integrins are heterodimeric receptors with α and β subunit, with several forms of these subunits resulting from different combinations. Cellular adhesion molecules (CAMs), members of the immunoglobulin superfamily expressed on the surface of vascular endothelial cells, are the natural integrin ligands. Both vascular cell adhesion molecule-1 (VCAM-1) and mucosal addressin cell adhesion molecule-1 (MAdCAM-1), which is specifically expressed on vascular endothelial cells of the gastrointestinal tract, are receptors for the α4 family of integrins. Disruption of integrin/CAM interactions blocks the recruitment of leukocytes across the endothelium and into inflamed parenchymal tissues. In CD, the interaction between α4β7 integrin and its endothelial receptor, MAdCAM-1, has been demonstrated as a relevant factor for the development of chronic intestinal inflammation (Arseneau and Cominelli, 2015).

In fact, the first compound that was developed that interfered with lymphocyte homing was natalizumab, a recombinant humanized IgG4κ monoclonal antibody that binds to the α4 subunit of two different integrins expressed on the surface of T and B cells: α4β1, responsible for homing of leukocytes to several inflamed, yet non-intestinal, tissues, such as skin, lung, and central nervous system, and α4β7, which has a specific role in the gut homing. By means of the bound to the α4 subunit, natalizumab inhibits α4-mediated adhesion of leukocytes to their receptors. The clinical effect of natalizumab in CD is most likely mediated by the inhibition of engagement between the α4β7 integrin and MAdCAM-1, expressed on the vascular endothelium in the actively inflamed gut. However, parallel actions on the α4β1 integrin/VCAM-1 pathway may still have a role for the inhibitory effects since VCAM-1 expression can be induced in submucosal vessels of the intestinal inflamed mucosa (Pagnini et al., 2017). Clinical efficacy of natalizumab has been demonstrated by three large phase III clinical trials: ENACT-1, ENACT-2, and ENCORE. The intent of the first two studies was to investigate the efficacy of natalizumab for the induction (ENACT-1) and maintenance (ENACT-2) of remission in moderate-to-severe CD patients (CDAI ≥ 220 and ≤450) in 142 centers. In the ENACT-1 trial, 905 CD patients were randomized 4:1 to natalizumab 300 mg/kg at 0, 4, and 8 weeks, or placebo, and response at week 10 was set as the primary endpoint. The induction study showed a significant difference in response and remission rate, but only in the subset of patients with high CRP levels (response = 58% vs. 45%, remission = 40% vs. 28%, p < 0.05 for both). In the ENACT-2 trial, patients from ENACT-1, who responded at week 10 and 12 (n = 339), were randomized 1:1 to maintenance therapy with natalizumab (300 mg/kg every 4 weeks) or placebo, and response (set as primary endpoint) and remission were evaluated at week 36. The results showed that in the treated group sustained clinical response and remission were significantly higher compared with patients in the placebo group (response = 61% vs. 28%, p < 0.001, and remission = 44% vs. 26%, p = 0.003) (Sandborn et al., 2005). Finally, the ENCORE trial confirmed the efficacy of natalizumab in the induction of remission in patients with high baseline CRP level (CDAI ≥ 220 and ≤450, and CRP > 2.87 mg/L) in a large study, which included 509 patients from 114 different centers. In the treatment group (three natalizumab infusions at week 0, 4, and 8) higher rates of response (48% vs. 32%, p < 0.001) and remission (26% vs. 16%, p = 0.002) at week 8 through week 12, compared to placebo, have been observed, with response and remission rates significantly higher in the treatment vs. placebo groups at every time point (4, 8, and 12 weeks) (Targan et al., 2007). Despite consistent positive results, the utilization of natalizumab has been strongly limited by safety issues. In fact, although rare, the occurrence of progressive multifocal leukoencephalopathy (PML), together with the development of a novel compound with a similar mechanism of action, but a more favorable safety profile, have contributed to the restricted use of natalizumab therapy for CD. Currently, natalizumab is approved for the treatment of multiple sclerosis worldwide, but is only approved for CD in the U.S. (and Switzerland), under a specific regulatory and distribution program (Pagnini et al., 2017).

Vedolizumab is a second-generation molecule that specifically interferes with leukocyte homing into the inflamed gut mucosa, and has consistently overcome safety issue of its predecessor, natalizumab. Vedolizumab has been approved for the treatment of IBD for the past few years, and represents the first biologic therapy specifically designed for a gastroenterological indication. Vedolizumab is a humanized IgG1 monoclonal antibody that specifically targets the α4β7 integrin, thereby preventing its binding to MAdCAM-1, exclusively expressed on the gut endothelium, with no effects on α4β1 integrin/VCAM-1 engagement, and consequently, no effect on leukocyte trafficking within the central nervous system. Such selectivity of action directly reflects on favorable drug safety profiles, with no cases of PML recorded thus far, and minimal adverse events confirmed in long-term follow-up studies (Colombel et al., 2017). The efficacy of vedolizumab to treat IBD patients has been demonstrated by large phase III studies, GEMINI I (for UC patients), and GEMINI II and III (for CD patients). In GEMINI I, 374 patients with active UC and unresponsive to treatment with either corticosteroids, immune modulators, or anti-TNF antibodies (nearly 40% of patients), were randomized (3:2) to receive vedolizumab (300 mg i.v. at weeks 0, 2, and 6) or placebo. The primary end point (clinical response defined by a decrease in Mayo score by at least three points from baseline, as well as no individual score greater than 1), evaluated at week 6, was achieved by 47% of patients when compared to those treated with vedolizumab vs. 26% of those patients receiving placebo (p < 0.0001). Secondary end-points, including clinical remission (17% vs. 5%, p = 0.001) and mucosal healing (41% vs. 25%, p = 0.001), showed significantly higher rates in vedolizumab- vs. placebo-treated patients. As follow-up, a total of 373 patients, including both patients who achieved clinical response in the randomized controlled trial and those who achieved clinical response during an open label trial, were re-randomized (1:1:1) to receive either placebo infusions or vedolizumab either every 4 or 8 weeks as maintenance therapy. The primary end-point (clinical remission at week 52) was achieved by 16%, 42%, and 45% of the placebo, as well as the 8- and 4-week vedolizumab groups, respectively (p < 0.0001 for both vedolizumab doses vs. placebo). Moreover, maintenance treatment with vedolizumab showed a durable clinical response (24%, 57%, and 52%, p < 0.0001), mucosal healing (20%, 52%, and 56%, p < 0.0001), durable clinical remission (9%, 20%, and 24%, p < 0.05 and p < 0.001, for 8- and 4-week vedolizumab groups vs. placebo, respectively), and corticosteroid-free remission (14%, 31%, and 45%, p < 0.05 and p < 0.0001), for 8- and 4-week vedolizumab groups vs. placebo, respectively, also through 52 weeks (Feagan et al., 2013). GEMINI II had a study design similar to that for GEMINI I: 368 patients with active CD, refractory to corticosteroids, immunomodulators, or anti-TNF antibodies (nearly 50% of patients), were randomized (3:2) to receive either vedolizumab (300 mg i.v. at weeks 0, 2, and 6) or placebo. Only one of the two primary end points for induction at week 6 was achieved; clinical remission rate (CDAI less than or equal to 150) was 15% in the treated group and 7% in the placebo group (p < 0.05), while no significant difference between groups was found for enhanced clinical response (CDAI decrease by 100 from baseline). Again, a total of 461 patients, including both patients who achieved clinical response in the randomized controlled trial of induction and patients who achieved clinical response during open label induction, were re-randomized (1:1:1) to receive placebo infusions, as well as vedolizumab, every 4 and every 8 weeks as maintenance therapy. The primary end point of clinical remission at week 52 was achieved in 39%, 36%, and 22% of patients assigned to maintenance vedolizumab infusions every 8 and 4 weeks, as well as those treated with placebo, respectively (p < 0.001 and p < 0.05 vs. placebo for 8- and 4-week groups). Moreover, enhanced clinical response (44%, 45%, and 30%, p < 0.05) for treatment groups vs. placebo, and corticosteroid-free remission (32%, 29% and 16%, p < 0.05) for treatment groups vs. placebo rates were significantly higher in maintenance vedolizumab compared to placebo (Sandborn et al., 2013). Finally, the GEMINI III study further explored the efficacy of vedolizumab for induction of remission in a particularly complicated set of patients, in which three quarters had previously failed anti-TNF therapy. In fact, 416 patients were randomized (1:1) to receive induction therapy with vedolizumab at 0, 2, and 6 weeks or placebo. The primary end point was clinical remission at week 6, but additional evaluation was performed at week 10. The results showed no statistically significant difference between treated and placebo groups; the week 10 analysis showed a trend for increased efficacy (15 vs. 12% at week 6 and 27 vs. 12% at week 10 in treated and placebo group, respectively) (Sands et al., 2014). Taken together, data from randomized control trials indicate that vedolizumab confirms the important therapeutic effect of blocking leukocyte homing to the gut mucosa, as already shown by the efficacy of natalizumab in CD. The important data on efficacy (in particular in UC patients) and its favorable safety profile have definitely launched vedolizumab for its use in treating IBD patients, both for anti-TNF-experienced patients (particularly for primary non-responders) and for naïve patients, especially those with absolute or relative contraindication for anti-TNF blockade. In fact, pool safety data from six trials including 2,830 patients have shown that vedolizumab is not associated with an increased risk of malignancy and/or serious or opportunistic infections, with infusion-related reaction reported in <5% of patients, and no cases of PML found (Colombel et al., 2017). Moreover, the favorable safety profile was confirmed by the results at 5 years of the GEMINI open-label extension study (Loftus et al., 2017). Data from GEMINI III indicated that in CD, the onset of the effect may be slower, specifically in TNF-experienced patients. Therefore, co-treatment with steroids, and/or a supplementary infusion at week 10 in the induction phase, may increase remission/response rates. Efficacy and safety of vedolizumab therapy has been further confirmed in real-life studies and observational studies, where even in CD patients, efficacy was better than in the registrative studies (Baumgart et al., 2016; Dulai et al., 2016; Amiot et al., 2017; Kopylov et al., 2017).

Some novel anti-integrin molecules, not yet approved for market distribution, have demonstrated efficacy for the treatment of IBD in clinical studies and deserve confirmation in large phase III trials. Abrilumab, a fully humanized monoclonal IgG2 antibody against α4β7, has demonstrated efficacy in the induction of remission in UC and CD (particularly in TNF-experienced CD patents) in two phase II studies. PF-00547659 (fully humanized MAdCAM-1 IgGk2 blocking antibody), AJM300 (oral small molecule targeting α4-integrin), and vercirnon (oral CCR9 small molecule antagonist) showed preliminary promising results, but need further evaluation for efficacy and safety (Arseneau and Cominelli, 2013; Park and Jeen, 2018). Among forthcoming anti-integrin molecules, etrolizumab appears to be the closest to market approval. It is a monoclonal IgG1 antibody against β7-integrin, a subunit common to α4β7 and αEβ7, whose ligand is E-cadherin, mainly expressed on epithelial cells. Thus, blocking β7-integrin, both lymphocyte trafficking into the gut and their retention in the intraepithelial compartment are prevented, with potentially less gut selectivity than vedolizumab, but hopefully higher efficacy. Efficacy of etrolizumab in inducing remission in moderate to severe UC patients have been demonstrated by a multi-centric, double-blind, placebo-controlled, randomized, phase II study, in which a total of 124 patients were randomized (1:1:1) to two different doses of subcutaneous etrolizumab or placebo. Primary end-point of clinical remission at week 10 was achieved in 8/39 (21%) of patients in etrolizumab 100 mg group (p = 0.004), in 4/39 (10%) of patients in etrolizumab 300 mg group (p = 0.048), and in no patient in placebo group, with no significant difference among groups for clinical response rate, and with serious adverse events recorded in 12%, 5%, and 12% in etrolizumab 100 mg, 300 mg, and placebo group, respectively (Vermeire et al., 2014). Large phase III studies further investigating efficacy and safety of etrolizumab in UC and CD patients are currently ongoing.

Another side of the leukocyte trafficking process that has been recently investigated for therapeutic purposes in IBD is the homing and egress of activated lymphocytes in lymph nodes. In particular, sphingosine-1 phosphate (S1P) receptor appears to have a relevant role in controlling lymphocyte trafficking to lymphoid organs, and blockade of that receptor arrests activated lymphocytes within lymph nodes, preventing their egress towards the gut (Nielsen et al., 2017). Fingolimod was the first S1P modulator approved for therapeutic purposes (in multiple sclerosis), but due to its unselective blockade of S1P, was characterized by important cardiovascular and hepatic side effects (Pelletier and Hafler, 2012). Ozanimod, an oral small molecule, selectively modulates S1P subtype 1 and 5 and has a more favorable safety profile. The molecule has shown preliminary proven efficacy for induction and maintenance of remission in UC patients in the double blind, placebo-controlled phase II TOUCHSTONE trial, in which 197 moderate 1 to severe UC patients were randomized (1:1:1) to treatment with ozanimod 0.5 mg/day, 1 mg/day, or placebo up to 32 weeks. In fact, primary end-point of clinical remission at week 8 was achieved in 11/67 (16%) of patients in the ozanimod 1 mg group (p = 0.048), 9/65 (14%) of patients in ozanimod 0.5 mg group (p = 0.14), and 4/65 (6%) of patients in placebo group, and clinical remission at week 32 was observed in 26%, 21%, and 6% in ozanimod 0.5 mg, 1 mg, and placebo group, respectively (p = 0.002 and p = 0.01 vs. placebo). At week 8, absolute lymphocyte counts reduction of 49% and 32% from baseline was observed in the ozanimod 1 and 0.5 mg group, respectively (Sandborn et al., 2016). Phase II and phase III trials that will further assess efficacy and safety of ozanimod, both in UC and CD patients, are still ongoing.

## Inhibitors of Pro-Inflammatory Cytokines

The first biologics that were developed to target pathogenic disease mechanism(s) were designed to inhibit the action of pro-inflammatory cytokines (for instance, TNF). In line with this original concept, new drugs blocking other pro-inflammatory cytokines, aside from TNF, have been developed as a therapy for IBD patients. IL-12/IL-23 represents the pathway most intensely investigated thus far. IL-12 and IL-23 are two cytokines of the IL-12 family that play an important role in the transition from innate to adaptive immune activation. Both are expressed by macrophages and dendritic cells activated by microbial stimulation, and promote acquired immunity through differentiation of naïve CD4^+^ cells into Th1 IFNγ-producing cells (IL-12) and Th17 cells (IL-23) that in turn activate a cascade of pro-inflammatory cytokines, such as IL-17, IL-6, and TNF (Iwakura and Ishigame, 2006). In particular, the activation of the IL-23/IL-17 axis appears of particular relevance to the pathogenesis of intestinal inflammation in IBD, as suggested from experimental observations (Becker et al., 2003; Tozawa et al., 2003). The first therapeutic agent of this class investigated in IBD is ustekinumab, a human IgG1k monoclonal Ab that binds the p40 subunit shared by both IL-12 and IL-23, thus preventing the interaction with their specific receptors on the surface of NK and T cells (Trinchieri et al., 2003). Ustekinumab, formerly approved for psoriasis and psoriatric arthritis, has recently been approved in Europe and then the U.S. for the treatment of CD patients. The efficacy of the drugs has been clearly demonstrated by three large multi-center, placebo-controlled phase III clinical studies reported in a single paper: UNITI-1, UNITI-2, and IM-UNITI (Feagan et al., 2016). The first two trials evaluated the induction of remission/response: CD patients with moderate-to-severe CD who failed or were intolerant to anti-TNF (UNITI-1; n = 741 patients), or naïve to anti-TNF therapy (UNITI-2; n = 628 patients) were randomized (1:1:1) to receive either ustekinumab at 130 mg IV, ustekinumab at 6 mg/kg, or placebo, with a primary end-point of clinical response at week 6, and a secondary end-point of clinical remission at week 8. Both primary (UNITI-1: 34.3%, 33.7%, and 21.15%, p < 0.003 vs placebo; UNITI-2: 51.7%, 55.5%, and 28.4%, p < 0.001 vs. placebo) and secondary end points (UNITI-1: 15.9%, 20.9%, and 7.3%, p = 0.003 and p < 0001 vs placebo; UNITI-2: 30.6%, 40.2%, and 19.6%, p = 0.009 and p < 0.0001 vs. placebo, respectively) were achieved in the treated groups. In the IM-UNITI trial, a total of 1,281 patients who responded to ustekinumab induction at week 8 (397 from UNITI-1 and UNITI-2 and 884 from an open label study) were randomized (1:1:1) to maintenance with ustekinumab (90 mg subcutaneous every 8 weeks, 12 weeks) or placebo, with a primary end-point of clinical remission at week 44, achieved by both treatment groups (remission rate: 53.1% and 48.8% in 8- and 12-weeks treatment, 35.9% in placebo group, p = 0.005 and p = 0.04, respectively). Safety profiles were similar between groups, and ustekinumab showed very limited immunogenicity (anti-drug antibodies at week 44 were found in 2.3% of patients). For the consistent efficacy rate and favorable safety profile, ustekinumab appears to be an important resource in the treatment of CD patients, both in TNF naïve and experienced patients, and particularly in patients with extraintestinal manifestations. Since most of the data come from registrative trials, post-marketing and real-life studies will hopefully confirm efficacy and safety data for this drug.

Considering the more prominent role of IL-23 over IL-12 blockade, novel drugs selectively targeting the IL-23/IL-17 axis have been investigated. Among the latter, MEDI2070 and risankizumab, monoclonal Abs that bind the p19 subunit of IL-23, have shown promising results in phase II trials, particularly in difficult-to-treat CD patients. In fact, in an induction study, MEDI2070 (700 mg IV at week 0–4) induced a significantly higher response rate than placebo in anti-TNF-experienced CD patients (49.2% vs. 26.7%, p = 0.01) (Sands et al., 2017). In addition, patients treated with risankizumab (200 mg IV and 600 mg IV) showed a significantly higher response (39% vs. 20.5%) and remission (30.5% vs. 15.4%) rate compared to placebo (Feagan et al., 2017).

Other molecules targeted at blocking pro-inflammatory cytokines are also being investigated, but results are preliminary. Tocilizumab, a fully humanized monoclonal antibody that blocks both soluble and membrane-bound IL-6, already approved for rheumatoid and juvenile arthritis, has shown potential efficacy in CD patients in a small pilot study (Ito et al., 2004), but no further investigation has been done. Very recently, results of a phase II trial evaluating PF-04236921, a subcutaneous monoclonal antibody blocking IL-6, has been published (ANDANTE I and II trial). In the induction study, 249 moderate-to-severe CD patients who had inadequate response to anti-TNFα were randomized 1:1:1:1 to placebo or 10, 50, or 200 mg of subcutaneous administration of PF-04236921 at day 1 and 28 (enrollment in the 200 mg group was prematurely stopped due to safety issue emerged in a trial in patients with systemic lupus erythematosus), and primary end-point was CDAI-70 response at weeks 8 and 12. In the open-label extension study, 191 patients received, after induction, PF-04236921 50 mg every 8 weeks for 48 weeks followed by 28 weeks of follow-up, in order to evaluate the safety of the treatment. In the induction study, PF-04236921 50 mg showed significant CDAI-70 response rate than placebo, both at weeks 8 and 12 (49.3 vs. 30.6% and 47.4 vs. 28.6%, respectively, p < 0.05 for both), and remission rate at week 12 was 27.4% in 50 mg treated group and 10.9% in placebo group (p < 0.05). Regarding safety, SAEs were reported in 30.4% of patients in the open-label extension study, and 6 patients in the induction study and 10 in the open-label extension study experienced abdominal abscess or perforation, complications at higher risk for the mechanism of action of the drug (Danese et al., 2019). Considering the results of this study, PF-04236921 appears a potentially useful treatment for difficult CD patients, but the lack of endoscopic efficacy data and the safety issue require further investigation in phase III trials.

Two phase II RCT trials investigated the potential efficacy of monoclonal antibodies blocking IL-13 (tralokinumab and anrunkinzumab) in UC patients, but primary end-points were not achieved (Danese et al., 2015; Reinisch et al., 2015). Quite surprisingly, besides the high efficacy of blocking IL-17 in other chronic inflammatory conditions, such as psoriasis, psoriatic arthritis, and ankylosing spondylitis, as well as the relevant role of IL-17 in CD pathogenesis and severity (Jiang et al., 2014), the IL-17 blockers, secukinumab and brodalumab, further worsened disease activity in CD patients in RCT trials (Hueber et al., 2012; Targan et al., 2016). Aside from multiple biological explanations, the negative results of IL-17 blockade make one reflect on the pleiotropic and complex molecular pathways underlying IBD, in which the same cytokine potentially possesses both protective and pathogenic effects on mucosal inflammation in different phases of disease, or within different compartments of the innate/acquired immune systems (Pagnini et al., 2010). Although similarities can be found with other chronic inflammatory conditions, such as rheumatoid arthritis, IBD represents a unique and peculiar pathologic entity, particularly considering the critical impact of the gut microbiome on disease pathogenesis. Therefore, translation of therapies, protocols, and specifics of management from other diseases need to be very cautiously addressed.

## Blockers of Downstream Cytokine Signaling Pathways

The Janus kinases (JAK), whose name derives from the double-faced Roman God, Janus, for the presence of two phosphate-transferring domains with opposite effects, are a group of heterodimeric intracellular enzymes that transduce the signaling generated by interaction between several cytokines and their cell surface receptors. Those interactions determine the activation of the JAK-STAT phosphorylation pathway, with the final result of nuclear transcription of effector proteins. There are four members of the JAK family [JAK1, JAK2, JAK3, and thyrosine kinase (TYK) 2] (Yamaoka et al., 2004). Since important pro-inflammatory cytokines, such as IFNγ, IL-2, IL-6, IL-12, and IL-23, utilize the JAK-STAT signaling system, blockade of its downstream activity has demonstrated therapeutic efficacy in several chronic inflammatory conditions, such as psoriasis (Papp et al., 2012), rheumatoid arthritis (Fleischmann et al., 2012), and, more recently, IBD. In particular, tofacitinib, a non-selective oral small molecule JAK inhibitor (that preferentially inhibits JAK1 and 3), has been recently investigated in CD and UC patients. In CD, tofacitinib failed to demonstrate significant response and remission rate over placebo both in induction and in maintenance phase II studies, but nonetheless showed a certain anti-inflammatory effect with a consistent reduction of C-reactive protein levels (Sandborn et al., 2014; Panés et al., 2017). More solid data have been shown by tofacitinib in phase III trials as a therapeutic agent in UC patients (Sandborn et al., 2017), and it has been recently authorized for marketing by the FDA and EMA for this indication. In two large induction trials, anti-TNF naïve (OCTAVE induction 1, n = 598) and experienced (OCTAVE induction 2, n = 541) UC patients were randomized 4:1 to tofacitinib (10 mg twice-a-day) or placebo. The drug-induced clinical remission at week 8 (primary end-point) was achieved at a significantly higher rate compared with placebo (18.5% vs. 8.2%, p = 0.007, and 16.6% vs. 3.6%, p < 0.001, in OCTAVE induction 1 and 2, respectively). A key secondary end-point of mucosal healing was achieved in both induction trials (31.3% vs. 15.6%, and 28.4% vs. 11.6%, in OCTAVE induction 1 and 2, p < 0.001 for both). In a maintenance trial, 593 patients who had clinical response to induction therapy were randomized (1:1:1) to tofacitinib (10 mg twice daily, 5 mg twice daily) or placebo. At week 52, the primary end-point of clinical remission was achieved in 40.6%, 34.3%, and 11.1%, of 10 mg, 5 mg, and placebo group, respectively (p < 0.001). The secondary end-point of mucosal healing was significantly higher in both treated groups comparing with placebo (45.7%, 37.4%, and 13.1%, p < 0.001). Besides these consistent efficacious results, some concerns about safety were raised by the OCTAVE trials. In fact, in the induction trials, overall infections, specifically serious infections, were higher in treated vs. placebo groups (23.3% vs. 15.6% in OCTAVE induction 1 and 18.2% vs. 15,2% in OCTAVE induction 2), and in the maintenance trial, a higher rate of overall infections (39.8%, 35.9%, and 24.2% in 10 mg, 5 mg, and placebo group, respectively) and herpes zoster infections (5.1%, 1.5%, and 0.5%) were recorded in treated vs. placebo groups. Moreover, more cases of non-melanoma skin cancers, cardiovascular events, and increased serum lipid levels have been observed across the three trials in treated patients. In order to overcome these safety issues, selective JAK1 blockers are now being investigated. Among the latter, filgotinib has shown the most promising results in CD patients in a phase II trial (FITZROY). In fact, a total of 174 CD patients with active disease, confirmed by a centrally read endoscopy, were randomized 3:1 to filgotinib 200 mg once a day or placebo for 10 weeks, and then re-randomized to filgotinib 100 mg/die, 200 mg/die, or placebo for an observational period of further 10 weeks. The primary end-point of clinical remission at week 10 was achieved by 47% of treated and 23% of placebo group (p < 0.01), and treated patients had a significant improvement of quality of life, but not statistically significant improvement of endoscopic activity has been observed, despite a trend for higher SES-CD 50%, endoscopic response, endoscopic remission, and deep remission in the treated group. Concerning safety, combining together the 20 weeks of the study, treatment-emergent adverse event rate was similar in treated and placebo group (75% and 67% in treated and placebo group, respectively), and serious treatment-emergent adverse events rate was 9% in treated and 4% in placebo group, with 3% of serious infection in treated group and none in placebo group. Interestingly, filgotinib was effective in TNFα-naïve (60% of remission rate at week 10) and -experienced patients (37%), and no significant serum lipid alterations were recorded (Vermeire et al., 2017).

## Other Mechanism(s) of Action

There are a few other drugs with mechanism(s) of action that are not applicable to the aforementioned categories, and that have been preliminary investigated as potential therapeutic options in IBD patients. For example, laquinimod is an oral small molecule that has demonstrated efficacy in the treatment of multiple sclerosis. Its mechanism of action has not yet been fully elucidated, but it is secondary to a shift in a regulatory phenotype of T-cells and reduction of pro-inflammatory cytokines (Varrin-Doyer et al., 2014). A phase II dose finding RCT showed that a lower dose of the drug (0.5 mg/day) has higher remission and response rates (48% and 55% respectively vs. 32% and 16% in the placebo group) in CD patients (D’Haens et al., 2015). Mongersen, an oral antisense oligonucleotide that binds SMAD7 mRNA, thus preventing the inhibition of TGFβ signaling, has previously shown consistent pre-clinical and clinical results in a phase II study, as well as effective impact on endoscopic activity in CD patients (Monteleone et al., 2015; Feagan et al., 2018). Unfortunately, the impressive results were not confirmed in a phase III trial, which was prematurely suspended after the interim analysis. As such, future development of drugs of this class is uncertain. A synthetic representation of the drugs evaluated in the current review is presented in **Table 1**.

**Table 1:** Main non anti-TNF pharmacological drugs that show beneficial effects in IBD therapy in randomized clinical trials.

Class	Drug	Route	Mechanism of action	Indication	Studies
Leukocyte trafficking	Natalizumab Vedolizumab Etrolizumab Ozanimod	IV IV IV oral	Antibody to α4 subunit Antibody to α4β7-integrin Antibody to β7-integrin Small molecule S1P 1-5 inhibitor	CD UC and CD UC and CD UC	Phase III—approved in US Phase III—approved Phase II
Inhibitors of pro-inflammatory cytokines	Ustekinumab MEDI2070 Risankizumab PF-04236921	IV/SC IV IV SC	Antibody to IL-12/IL-23 (p40) Antibody to IL-23 (p19) Antibody to IL-23 (p19) Antibody to IL-6	CD CD CD CD	Phase III—approved Phase II Phase II Phase II
Blockers of downstream cytokine signaling pathways	Tofacitinib Filgotinib	oral oral	Small molecule JAK blocker Small molecule JAK1 blocker	UC CD	Phase III—approved Phase II
Other pathways	Laquinimod Morgensen	oral oral	Small molecule active on T-cells Antisense nucleotide of SMAD7	CD CD	Phase II Phase II—phase III suspended

IV, intravenous; SC, subcutaneous; CD, Crohn’s disease; UC, ulcerative colitis.

## Discussion

Published studies and real-life experiences indicate that standard biologic therapy with anti-TNF blockers, which often remains the first-line therapy for moderate-to-severe IBD patients not responding to conventional therapy, has several drawbacks and the majority of patients either still do not respond or lose response over time. This issue has pushed the field towards research and characterization of novel drugs that are potentially useful for the treatment of IBD, with different mechanisms of action from TNF blockade. Among these drugs, three (i.e., natalizumab, vedolizumab, and ustekinumab) are already available for clinical use and one (tofacitinib) has received approval for market distribution, while the other molecules are still in the pipeline. As a consequence, the efficacy data are mostly preliminary and mainly coming from registrative or RCT trials, in which the clinical conditions are often not comparable to reality. Even more relevant, safety data are absolutely preliminary, since registry data and post-marketing surveillance, which are fundamental tools for the identification and evaluation of drug safety, are mostly lacking for novel molecules. Nonetheless, the expansion of the drug armamentarium for the treatment of IBD patients in recent years is remarkable, as well as the positive perspectives for the near future, considering the relative paucity of effective IBD drugs compared to other chronic inflammatory conditions.

At present, a number of relevant questions are raised, and several issues need to be addressed, including: are the new drugs really improving the efficacy of treating IBD patients? Did they offer a gain in remission/response rate in naïve and TNF-experienced IBD patients? Are the drugs better than the “established” anti-TNF blockers? Perhaps the more correct way to respond to these inquiries would be to evaluate head-to-head trials, directly comparing different drugs to each other. Unfortunately, results of these trials are not yet available. A phase III multicenter clinical trial directly comparing vedolizumab and adalimumab in UC patients has, to date, recruited 770 patients and results are pending for 2019 (ClinicalTrials.gov Identifier: NCT02497469). Furthermore, a phase III study comparing etrolizumab vs. adalimumab in UC patients (ClinicalTrials.gov Identifier: NCT02171429) and ustekinumab vs. adalimumab in CD patients (ClinicalTrials.gov Identifier: NCT03464136) are still in the process of recruiting patients. Since results of these studies are not yet available, the only surrogate data we have for drug comparison studies derive from indirect statistical evaluation of different trials, by means of network meta-analysis. To date, 10 studies have been published, among which 4 evaluated trials in UC patients (Danese et al., 2014; Vickers et al., 2016; Bonovas et al., 2018; Singh et al., 2018), 4 in CD patients (Singh et al., 2014; Stidham et al., 2014; Hazlewood et al., 2015; Pagnini et al., 2018), and 2 studies both CD and UC patients (Cholapranee et al., 2017; Mao et al., 2017) have been performed. Together, the results of these studies further confirm the efficacy of treatments vs. placebo, both in naïve and TNF-experienced patients, but failed to identify a drug clearly superior to the others. Moreover, such results need to be interpreted with great caution, since heterogeneity among studies may profoundly affect meta-analysis results.

A further speculation that one could make is: now that drugs with multiple mechanism(s) of action are competing in the market for IBD treatment, how can we improve the efficacy of such therapies? Probably the best approach is to attack from several fronts by improving the selection of patients by identification of pre-treatment features predictive of response to a specific drug, and evaluating the possibility of multi-drug co-treatments. In fact, the concept of personalized medicine for the treatment of IBD patients has been recently explored. Recognizing clinical and/or molecular markers predictive of response to specific drugs may be of paramount importance in the near future, when more drug options become available. This could be relevant for both the first drug of choice in naïve patients, when an effective therapeutic strategy is more likely to positively impact long-term outcome, and for patients who have already failed a first line of therapy, where a specific second-line drug may guarantee a higher response rate in this difficult-to-treat subset of patients. Most of the research in this field of investigation focuses on predictors of response to anti-TNF therapy. Considering clinical characteristics, some studies have demonstrated that, in CD patients, young age, isolated colitis, and elevated CRP levels are predictors of response to anti-TNF therapy, while smoking and disease duration more than 2 years are predictors of non-responders (Louis et al., 2002; Parsi et al., 2002; Arnott et al., 2003; Siegel and Melmed, 2009). More recently, Barrè et al. performed a literature review on predictors of response to vedolizumab and ustekinumab. The authors found that severe disease, prior anti-TNF exposure, was a negative predictor of vedolizumab response, while ileocolonic disease, no prior surgery, and an uncomplicated phenotype were associated with a better response to ustekinumab in CD (Barre et al., 2018). Focusing on gene expression profiles, Arijs et al. (2010) identified a five gene set (i.e., TNFAIP6, S100A8, IL11, G0S2, S100A9) that discriminated with 100% accuracy patients with CD colitis as responders vs. non-responders to infliximab, while no predictive genes were found in CD ileitis. The same authors identified top five genes (i.e., osteoprotegerin, stanniocalcin-1, prostaglandin-endoperoxide synthase 2, IL-13 receptor α2, IL-11) differentially expressed in UC patient responders and non-responders to infliximab (Arijs et al., 2009). Moreover, utilization of molecular imaging, such as single photon emission computed tomography (SPECT) and positron emission tomography (PET), have been evaluated for prediction of response to anti-TNF therapy. Van den Brande et al. (2007) demonstrated that (99 m) technetium (Tc)-annexin V SPECT could discriminate infliximab responders vs. non-responders in a murine model and in CD patients. In another study, CD patients with high numbers of membrane-bound TNF immune cells, detected by topical antibody administration, showed significantly higher short-term response rates to anti-TNF therapy compared with patients with low cell counts (Atreya et al., 2014). Interestingly, two very recently published elegant studies investigated the occurrence of specific inflammatory pathways in IBD patients associated with a low response to anti-TNF therapy. West et al. demonstrated high tissue levels of oncostatin M, a member of the IL-6 cytokine family with potent pro-inflammatory activity, in an animal model of anti-TNF resistant intestinal inflammation and in anti-TNF non-responder IBD patients from two cohorts of phase III clinical trials. The authors propose OMS as a potential biomarker and therapeutic target in IBD that may have an important role in anti-TNF resistant patients (West et al., 2017). Gaujoux et al. observed a significant increase, pre-treatment, in plasma cells from biopsy samples of anti-TNF non-responder patients, which was coupled to an increase in triggering receptor expressed on myeloid cells 1 (TREM-1) and the chemokine receptor type 2 (CCR2)–chemokine ligand 7 (CCL7) axes. In addition, the authors showed that pre-treatment downregulation of TREM expression in peripheral blood of CD patients accurately predicts non response to anti-TNF therapy with an AUC of 94%, thus proposing systemic TREM-1 expression as a non-invasive diagnostic marker of non-response to anti-TNF therapy at baseline (Gaujoux et al., 2018). In addition, interesting data have been obtained from an etrolizumab phase II trial and from retrospective analysis. In fact, higher levels of granzyme A and integrin αE mRNA expression in colon tissues could discriminate patients who are more likely to respond to the drug (Tew et al., 2016). Moreover, serum pre-treatment concentration of IL-22 has been proposed as predictor of response to the IL-23 blocker MEDI2070, and cytokine level above 15.6 pg/ml has been associated with higher response rate, while clinical outcome similar to placebo has been observed in CD patients with IL-22 serum level below that threshold value (Sands et al., 2017). Recently, differences in microbiome composition at baseline have been preliminarily investigated as a potential biomarker predictor of response to infliximab (Shaw et al., 2016) and vedolizumab (Ananthakrishnan et al., 2017), but more data are needed at this time to properly evaluate its predictive value.

The availability of drugs interfering with different molecular inflammatory pathways may bear the attractive concept that contemporaneous block of multiple pathways results in high efficacy, but safety issues need to be considered. A recent systematic review showed limited benefit for combination biologic therapy in rheumatoid arthritis, while data in IBD patients, coming from a few case reports, suggest potential benefit (Hirten et al., 2015; Yzet et al., 2016; Bethge et al., 2017; Fischer et al., 2017). The only explorative study for combination therapy in CD is a multi-center randomized clinical trial primarily evaluating the safety and tolerability of three natalizumab infusions in 79 CD patients with active disease, despite infliximab therapy. Besides the small number of patients and the fact that the study was not designed for efficacy evaluation, a trend for higher response rates in the natalizumab + infliximab group was observed compared to placebo + infliximab, and safety evaluation was reassuring (Sands et al., 2007). An open label prospective study, EXPLORER (Clinical Trials.gov Identiﬁer: NCT02764762), aimed at determining the effect of triple combination therapy with vedolizumab, adalimumab, and methotrexate on endoscopic remission in moderate-to-severe CD patients, stratiﬁed at higher risk for complications, is also currently underway.

In conclusion, in recent years, novel drugs with different mechanism(s) of action are likely to expand the physician’s armamentarium to treat IBD patients. Such novel drugs should confirm the positive results from registrative trials in real-life settings, where difficult-to-treat patients are more frequent and ideal conditions of the trials are not considered. At the same time, the increased availability of therapeutic options represents a great opportunity and challenge for IBD specialists. In fact, the correct stance and implementation for use of available novel drugs, together with an increased ability in patients’ selection and therapeutic tailoring, will hopefully lead to more effective therapies, as well as increased safety, for the treatment of IBD patients.

## Author Contributions

CP conceived and wrote the manuscript. TTP wrote and edited the manuscript. FC conceived, wrote, and edited the manuscript.

## Conflict of Interest Statement

The authors declare that the research was conducted in the absence of any commercial or financial relationships that could be construed as a potential conflict of interest.
